# Genomic Predictions of Phenotypes and Pseudo-Phenotypes for Viral Nervous Necrosis Resistance, Cortisol Concentration, Antibody Titer and Body Weight in European Sea Bass

**DOI:** 10.3390/ani12030367

**Published:** 2022-02-02

**Authors:** Sara Faggion, Daniela Bertotto, Valentina Bonfatti, Matteo Freguglia, Luca Bargelloni, Paolo Carnier

**Affiliations:** 1 Department of Comparative Biomedicine and Food Science (BCA), University of Padova, 35020 Padua, Italy; valentina.bonfatti@unipd.it (V.B.); luca.bargelloni@unipd.it (L.B.); paolo.carnier@unipd.it (P.C.); 2 Valle Cà Zuliani Società Agricola S.r.l., 48017 Ravenna, Italy; matteo.freguglia@vallecazuliani.it

**Keywords:** *Dicentrarchus labrax* L., genomic prediction, EBV, GWAS, nodavirus, cortisol, antibody titer

## Abstract

**Simple Summary:**

Selective breeding programs based on genomic data are still not a common practice in aquaculture, although genomic selection has been widely demonstrated to be advantageous when trait phenotyping is a difficult task. In this study, we investigated the accuracy of predicting the phenotype and the estimated breeding value (EBV) of three Bayesian models and a Random Forest algorithm exploiting the information of a genome-wide SNP panel for European sea bass. The genomic predictions were developed for mortality caused by viral nervous necrosis, post-stress cortisol concentration, antibody titer against nervous necrosis virus and body weight. Selective breeding based on genomic data is a possible option for improving these traits while overcoming difficulties related to individual phenotyping of the investigated traits. Our results evidenced that the EBV used as a pseudo-phenotype enhances the predictive performances of genomic models, and that EBV can be predicted with satisfactory accuracy. The genomic prediction of the EBV for mortality might also be used to classify the phenotype for the same trait.

**Abstract:**

In European sea bass (*Dicentrarchus labrax* L.), the viral nervous necrosis mortality (MORT), post-stress cortisol concentration (HC), antibody titer (AT) against nervous necrosis virus and body weight (BW) show significant heritability, which makes selective breeding a possible option for their improvement. An experimental population (N = 650) generated by a commercial broodstock was phenotyped for the aforementioned traits and genotyped with a genome-wide SNP panel (16,075 markers). We compared the predictive accuracies of three Bayesian models (Bayes B, Bayes C and Bayesian Ridge Regression) and a machine-learning method (Random Forest). The prediction accuracy of the EBV for MORT was approximately 0.90, whereas the prediction accuracies of the EBV and the phenotype were 0.86 and 0.21 for HC, 0.79 and 0.26 for AT and 0.71 and 0.38 for BW. The genomic prediction of the EBV for MORT used to classify the phenotype for the same trait showed moderate classification performance. Genome-wide association studies confirmed the polygenic nature of MORT and demonstrated a complex genetic structure for HC and AT. Genomic predictions of the EBV for MORT could potentially be used to classify the phenotype of the same trait, though further investigations on a larger experimental population are needed.

## 1. Introduction

In fish species, improvements in selection accuracy due to genomic predictions and benefits of genomic selection over pedigree-based methods have been documented both in simulation studies [1] and real data from applied selective programs for growth and disease resistance [2,3,4,5,6,7]. Disease outbreaks may have important impacts on aquaculture, threatening the survival of farmed animals, interfering with the productivity and sustainability of intensive systems and consequently, causing significant economic losses.

Selective breeding applied to aquaculture for economically important traits is a relatively young practice compared to plant and livestock species [8], and its development has mainly followed the industrialization and growth of the aquaculture sector [9,10]. Currently, beyond biosecurity and management practices, selective breeding for disease resistance is viewed as a feasible and sustainable approach to prevent and control damages inflicted by disease outbreaks in aquaculture farms [8,11].

For the European sea bass (*Dicentrarchus labrax* L.) industry, one of the major threats is viral nervous necrosis (VNN), and interest in enhancing genetic resistance to VNN has recently greatly increased [12]. Low-to-moderate heritability (*h*^2^) estimates for VNN resistance [2,13,14,15] indicate the presence of a genetic basis for this trait, and thus, opportunities to develop selective breeding approaches.

In a previous paper [13], we estimated the moderate heritability (*h*^2^ = 0.28–0.39) of antibody titer against nervous necrosis virus (NNV) antigens in sea bass and a negative genetic correlation (r_a_ = −0.39) of the antibody titer with VNN mortality. These results suggested that the NNV antibody titer could be used as an indicator trait to enhance VNN resistance.

Low stress-sensitive fish have shown an increased ability to cope with environmental challenges typical of intensive systems, and they have also shown better performance traits (efficiency, productivity and resilience [16]). Yet, though a relationship between the stress response and disease resistance has been hypothesized [17], use of the serum post-stress cortisol concentration—the most relevant physiological stress-response measure [18]—as an indicator trait for VNN resistance is questionable due to the lack of a genetic relationship with mortality [13]. In sea bass, significant additive genetic variation in the post-stress cortisol level has been reported [13,19,20], making selective breeding for this trait feasible.

Traditional selective breeding approaches make use of estimated breeding values (EBV) predicted from individual phenotypes of the breeding candidates or their relatives through additive genetic relationships between animals. Such selection programs are currently most commonly adopted by European breeding companies [9,10]; their application ensures the achievement of relevant genetic gains for many economically important traits.

Routine individual phenotyping of breeding candidates or their relatives for complex traits such as VNN resistance, antibody titer or cortisol concentration is either difficult or unfeasible, as well as being costly and time-consuming. For this reason, genetic improvement of such traits cannot be achieved efficiently with traditional methods [21,22]. Yet, given the availability of new genomic tools and techniques enabling the development of high-density marker arrays [23], genomic selection procedures may be implemented and might be greatly beneficial [8]. In a framework of genomic selection, the phenotypic records and the genotypes of a reference population, which are representative of the genetic characteristics of the breeding candidates, are used to estimate the SNP effects on the traits of interest, to predict the genetic merit of breeding candidates that are subjected to genotyping alone [24].

This study aimed to investigate the performance of genomic models by making use of the individual genotypes of 16,075 genome-wide SNPs as predictors of the VNN mortality, post-stress cortisol concentration, NNV antibody titer and body weight, predicting both trait phenotypes and pseudo-phenotypes (EBV). The performance of those predictions in the classification of VNN mortality was compared with the performance of classification based on EBV estimated from full information including the individual phenotype, or from information of full- and half-sibs alone.

## 2. Materials and Methods

### 2.1. Experimental Fish: Production, Rearing and NNV Challenge Test

The procedures for the production, rearing and NNV challenge test of the experimental fish were detailed in [13]. Briefly, artificial fertilization was performed using a commercial NNV-free ELISA-tested breeding stock. The broodstock was of Atlantic (~25%) and West-Mediterranean (~75%) origin. The mating scheme was based on an incomplete factorial design where four groups of five sexually mature females were mated to four groups of eight sexually mature males, with a sire-to-dam mating ratio of 8 to 5. The mean weights (±SD) of the females and males were 6.45 ± 0.62 kg and 5.18 ± 0.66 kg, respectively. Before being transferred to the experimental facility of the Istituto Zooprofilattico Sperimentale delle Venezie (IZSVe, Legnaro, Italy) for NNV infection, the fish were reared in a sea cage.

The challenge test with NNV was carried out using 652 randomly chosen fish at an age of 548 d post-hatching, with an average body weight (BW) of 146.4 g (CV% 29.18). Before being infected by intraperitoneal injection of 0.1 mL viral suspension (RGNNV 283.2009, batch 7/16; 10^8.30^ TCID_50_ per mL), the fish were subjected to a stress test (acute stress, confinement at high density, ~80 kg/m^3^ for 10 to 14 min), anesthetized (30 ppm of MS-222), individually tagged with passive integrated transponders (PIT-tag) and individually blood-sampled to perform an indirect ELISA assay and cortisol analysis by radioimmunoassay (RIA).

The fish were distributed between three 2500 L and three 380 L closed-system tanks. After infection, they were checked three times a day to record clinical signs of VNN and mortality (MORT). Dead fish were classified as 1. The experiment ended at 29 d post-challenge and a cumulative survival rate of 52% was recorded. All fish that were alive at day 29 were euthanized with an overdose of anesthetic (MS-222) and classified as survivors (MORT = 0). Tissue samples (muscle and fin) were collected from each fish (dead or survived) and preserved in absolute ethanol for genomic DNA extraction. The experimental protocol was evaluated and approved by the Italian Ministry of Health (Law decree 26/2014 art. 31; permission number: 975/2016-PR of 13/10/2016).

### 2.2. Assessment of Phenotypes for Antibody Titer against NNV and Cortisol Concentration

An indirect ELISA assay for antibodies against NNV and cortisol extraction and measurement were carried out as described in [13]. Briefly, an indirect ELISA assay was performed at the Istituto Zooprofilattico Sperimentale delle Venezie (IZSVe, Legnaro, Italy) on the blood serum collected from each fish before infection, following the protocol of [25,26].

To do so, 5 µL 10^7^ TCID_50_ of virus preparation (pAb 283, rabbit polyclonal antiserum recognizing VERv, raised against strain 283/I09–RGNNV) diluted in 95 µL 0.05 M carbonate–bicarbonate buffer (pH 9.4) was employed to coat 96-well polystyrene plates (Maxisorp, Nunc, Roskilde, Denmark). After an incubation period (4 °C) and washing procedure (50 mM Tris-HCl at pH 7.4 containing 0.05% Tween-20 and 0.15 M NaCl, TTN), the remaining sites were blocked with 3% BSA in TTN (TBT). Plates were incubated at room temperature and washed again, then incubated (4 °C) with various dilutions of pAb 283 diluted in TBT. Sera were analyzed in duplicate wells. The wells were carefully washed with TTN and then incubated for 2 h at 4 °C with a 100 µL dilution of horseradish peroxidase (HRP)-conjugated goat IgG fraction to rabbit IgG (GAR-HRP). The wells were carefully washed and the reaction was developed with 100 µL/well of 0.04% o-phenylenediamine in 50 mM phosphate-citrate buffer (pH 5.0) containing 0.001% H_2_O_2_. The reaction was allowed to proceed for 20 min at 22 °C, stopped with 50 µL of 3 N sulfuric acid, and the absorbance was read at 450 nm with an automatic plate reader (Labsystems Multiskan MS, Thermo Fisher Scientific, Waltham, MA, USA). The ELISA optical density (OD, 450 nm) values’ sample-to-positive ratio (S/P ratio) was applied to evaluate the status of the sample. The trait was abbreviated as AT (antibody titer).

Cortisol was extracted from the blood serum, following the protocol by [27], and the concentration of cortisol in each sample was analyzed through solid-phase radioimmunoassay [28]. Radioactivity values counted on a β-counter (Top-Count NXT, Perkin Elmer Life and Analytical Sciences, Waltham, MA, USA) were analyzed with GraphPad Prism 5.0 software (La Jolla, CA, USA) to determine the cortisol concentration of each sample (expressed as pg/well), which was later converted to ng per ml of serum, normalized through square root transformation and abbreviated as SRHC.

### 2.3. Genotyping, Parentage Assignment and Pedigree Reconstruction

All the phases of genotyping, parentage assignment and pedigree reconstruction were detailed in [13]. Genomic DNA was extracted from muscle or fin samples of the experimental fish (N = 652) and their parents (N = 52) using the commercial Invisorb^®^ Spin Tissue Mini Kit and Invisorb^®^ DNA Tissue HTS 96 Kit (Invitek, STRATEC Biomedical, Germany), which was then quality-checked and quantified. A 2b-RAD library was constructed for each individual following the protocol by [29] with minor modifications. Individual libraries were pooled into equimolar amounts (52 for parents, 96–97 for offspring). Pooled libraries were analyzed with an Agilent 2100 Bioanalyzer (Agilent Technologies, Santa Clara, CA, USA) and then sequenced on an Illumina HiSeq4000 platform with a 50 bp single-read module at Fasteris SA (Plan-les-Ouates, Switzerland) and UC Davis (Davis, CA, USA), along with demultiplexing and a first quality-check of the raw data. After filtering and trimming, the fragments were elaborated with STACKS software 2.0 [30], firstly mapping the trimmed reads against the European sea bass genome [31] (http://seabass.mpipz.mpg.de/DOWNLOADS/dicLab1_scaffold.fasta, accessed on 17 May 2018) and then secondly identifying SNPs. The results were filtered to exclude loci shared by less than 75% of the individuals, resulting in a dataset of 18,097 SNP genotypes per fish. Some SNPs were discarded based on a minor allele frequency (MAF) of lower than 1%, genotype frequencies deviating from the expected Hardy-Weinberg equilibrium frequencies (*p* < 0.001) and the presence of a missing genotype in more than 15% of the individuals. Missing genotypes for the remaining SNP were imputed using the FImpute software [32]. The final number of available SNP genotypes per animal was 16,075. Two individuals in the experimental population were discarded because of low genotyping quality.

Parentage assignment and pedigree reconstruction were performed using the software CERVUS 3.0 [33,34] and the R package sequoia [35]. The experimental fish were assigned to a unique parental pair: 41 parents were assigned to 22 dummy parental pairs (14 grandfathers and 8 grandmothers), 3 to 3 single dummy parents (1 grandfather and 2 grandmothers) and 8 remained unassigned. The overall number of full-sibs families was 136 as the progeny of 19 dams and 30 sires (2 sires and 1 dam were not assigned to any individual in the challenged sample).

### 2.4. Genome-Wide Association Study

A genome-wide association study (GWAS) was performed on MORT, SRHC and AT to test the association between phenotypes and SNP genotypes using the R package GASTON [36]. Due to the binary nature of MORT, inference of the association was based on solutions of the following mixed logistic regression model:

(1) η=1μ+Xb+Wu+zα

where η is a vector of logits, defined as $\log\left[\frac{\pi_{i}}{1-{\;\pi }_{i}}\right]$ (where $\pi_{i}$ is the probability that the phenotype is 1 (dead) for animal i), μ is the model intercept, **1** is an all-ones vector, b is a vector of unknown fixed parameters (fixed effects) due to the tank, **X** is an incidence matrix relating η to **b**, u is an unknown vector of random animal additive genetic effects assumed to be $N\left(0,\;G\sigma_{g}^{2}\right)$ (where N( ) indicates a normal probability density function), **G** is the genomic relationship matrix and $\sigma_{g}^{2}$ is the additive genetic variance, **W** is an incidence matrix relating η to u, α is the unknown allele substitution effect of a single SNP and **z** is a vector of marker genotypes, coded as the number of copies of the minor allele, relating η to α.

For SRHC and AT, hypothesis testing was carried out on the basis of solutions to the following linear mixed model:

(2) y=1μ +Wu+zα+e

where y is a vector of phenotypes, **1**, µ, W, u, z and  α have the meanings described for the logistic regression mixed model and e is a vector of random residuals assumed to be $N\left(0,\;I\sigma_{e}^{2}\right)$ (where **I** is an identity matrix of appropriate order and $\sigma_{e}^{2}$ is the residual variance). Estimates of $\sigma_{g}^{2}$ and $\sigma_{e}^{2}$ used to solve the model originated from [13].

The genome-wide significance threshold was set to *p* = 0.05/N where N is the total number of genetic markers considered in the association analysis [37], thus applying a Bonferroni correction of the significance cut-off to deal with the multiple testing problem. Manhattan plots of GWAS results were obtained with the R package qqman [38].

### 2.5. Prediction of Breeding Values

Animal additive genetic effects (i.e., breeding values, EBV) for MORT, BW, SRHC and AT were predicted using mixed animal models. A Bayesian approach employing Monte-Carlo Markov Chain (MCMC) and Gibbs sampling methods was implemented using the software TM [39]. For MORT, the variation in the phenotypic expression of the trait (y = 0 for the fish that were alive at the end of the NNV challenge test, y = 1 for the dead ones) was assumed to be dependent on the value of an unobservable and normally distributed latent variable λ, the liability [40], and was investigated through the use of a threshold model. Under such a model, the probability for the observed categorical phenotype is as follows:

(3) $$P(y=0|x)=P\left(\lambda \leq \lambda_{t}|x\right),\;P(y=1|x)=P\left(\lambda >\lambda_{t}|x\right)$$

where x is a set of explanatory variables, which implies that mortality is observed when the liability exceeds a particular threshold level $\lambda_{t}$. By using the cumulative standard normal density function, such probabilities can be obtained as follows:

(4) $$P\left(\lambda \leq \lambda_{t}|x\right)=\Phi (\lambda_{t}-\lambda |x),\;P\left(\lambda >\lambda_{t}|x\right)=1-\Phi (\lambda_{t}-\lambda |x)$$

where Φ( ) denotes the cumulative standard normal density function (probit function).

In our study, the liability was modeled as:

(5) λ=1μ+Xb+Wu+e

where λ is a vector of underlying liabilities, μ is the model intercept, **1** is an all-ones vector, b is a vector of unknown fixed parameters (fixed effects) due to the tank, **X** is an incidence matrix relating λ to **b**, u is a vector of unknown random animal additive genetic effects and e is a vector of random residuals. Additive genetic effects and the residuals were assumed to be distributed as N$\left(0,A\sigma_{g}^{2}\right)$ and $N\left(0,I\sigma_{e}^{2}\right)$, respectively, where N( ) indicates a normal probability density function, **A** is the numerator relationship matrix, $\sigma_{g}^{2}$ is the additive genetic variance, **I** is an identity matrix of appropriate order and $\sigma_{e}^{2}$ is the residual variance. In the Bayesian analysis, the liability vector was treated as a nuisance parameter and was integrated out in the Gibbs sampler. Solutions for model parameters (μ, b and u) were obtained using the **A** matrix computed from the reconstructed pedigree, the estimate of $\sigma_{g}^{2}$ reported in [13] and by setting $\lambda_{t}=0$ and $\sigma_{e}^{2}=1$.

For BW, SRHC and AT, the prediction of EBV was based on the following linear mixed model:

(6) y=1μ +Wu+e

where y is a vector of observed phenotypes and **1**, µ, W, u and  e  have the meanings described for the threshold mixed model. Assumptions on the probability distribution of u and e were as in the model for the liability of MORT. The variance components of random effects, used to solve the model, were those reported in [13].

The prior densities used in all Bayesian analyses were uniform densities for the fixed effects and normal densities for the additive genetic and residual effects. The Gibbs sampler was run using 100,000 iterations with a burn-in of 5000 iterations and a thinning interval of 10 samples.

The predicted EBVs were used as “pseudo-phenotypes” in the development of genomic predictions described in the next section of the paper and will be referred to as EBV_FULL_ to indicate that they were estimated using all available information, which included the observed phenotype of the individual and those of its full- and half-sibs.

### 2.6. Genomic Predictions

#### 2.6.1. Bayesian Regression Models

Genotypes of the 16,075 SNPs were used as predictors of the phenotypes or EBV_FULL_ for the investigated traits by means of three Bayesian regression models fitted to the data using the BGLR package in the R software [41]. These models differed in the prior probability density assumed for the genotype effects. For Bayes B (BB) and Bayes C (BC) [42], the prior density for genotype effects was a finite mixture density of a point of mass at zero and a scaled-t or Gaussian slab, respectively. The third model was a Bayesian Ridge Regression (BRR) model [24] using, as a prior for genotype effects, the Gaussian density. Each model had the following general form:

(7) $$y=1\mu +\overset{S}{\underset{j=1}{\sum }}x_{j}b_{j}+e$$

where y is a vector of observed phenotypes or EBV_FULL_ for the trait under investigation, μ is the model intercept, **1** is an all-ones vector, $x_{j}$ is a vector of genotypes at SNP j, coded as the number of copies of the minor allele (0, 1 or 2), $b_{j}$ is the allele substitution effect for the minor allele at SNP j (to be estimated), S is the total number of SNP (S = 16,075) and e is a vector of random residuals (which was assumed to be $N\left(0,I\sigma_{e}^{2}\right)$, where N( ) indicates a normal probability density function), **I** is an identity matrix of appropriate order and $\sigma_{e}^{2}$ is the residual variance. In the analysis of the phenotype for MORT, the probit function was used as a function to link the probability of each of the categories (0 = alive, 1 = dead) to the linear predictor, to properly account for the binary nature of the trait, while for identification purposes, the residual variance was set as equal to 1. Each Bayesian analysis was carried out generating a single Gibbs chain of 800,000 samples, with a burn-in of 10,000 iterations and a thinning interval of 50 samples.

#### 2.6.2. Random Forest Algorithm

Random Forest (RF) [43] is a machine learning method for classification and regression, also defined as an “ensemble learner”. Briefly, the algorithm operates by aggregating a multitude of “base learners” (decision trees) to create the ensemble (the forest). Each decision tree is grown using two-level randomization in the learning process: (a) a bootstrapped sample of the training data is used to grow the tree and (b) a subset of *m* variables is randomly selected from the set of *p* available predictors and evaluated to identify the rule splitting the parent node into two smaller daughter nodes. The splitting rule is based on a single variable belonging to the selected subset of *m* predictors.

We finetuned the RF algorithm by comparing the error rate in the prediction or classification of the response variable (phenotype or EBV_FULL_) for different values of the following parameters: the number of trees to grow (20,000, 80,000 or 160,000) and the minimum size of terminal nodes (5, 10 or 20). The predictors (*p* = 16,075) were the genotypes coded as described previously. The number *m* of predictors randomly sampled for node splitting was set as the default (i.e., $\sqrt{p}$ for classification and *p*/3 for regression) [44]. It was observed that 80,000 trees and a terminal node size of 10 were enough to stabilize the prediction and classification errors. These values were then used in the RF classification of MORT phenotypes and in the RF regression analysis of phenotypes of continuous traits and of all EBV_FULL_. Analyses were implemented using the randomForestSRC package [45] in the R software.

#### 2.6.3. Assessment of Model Performance in Prediction and Classification

The metrics used to predict and classify the performance of the Bayesian models and RF algorithm were computed in a set of 16 independently-generated five-fold cross-validations (CVs). In each CV, the data (N = 650) were randomly split into five equally-sized data segments. Four of these data segments (80% of the data) were used as a training set to obtain solutions for allele substitution effects, whereas the remaining segment served as a test set. In the test set, the phenotypes or EBV_FULL_ for the trait under study were set to missing, and they were predicted or classified from the SNP genotypes using the solutions of the Bayesian models or the RF obtained in the analysis of the training data. Predictions and classifications for each test set were saved, and at the end of the CV, aggregated with those of the other test sets to compute performance metrics.

When predicting the phenotype of continuous traits (BW, SRHC and AT) or the EBV_FULL_ of any trait, the Pearson product-moment correlation (*r*) between the observed and predicted values was computed as a measure of prediction accuracy. The accuracy in the prediction of the additive genetic component of the phenotype (*r*_adj_) was derived from the prediction accuracy *r* as follows:

(8) $$r_{\mathrm{adj}}=r/\sqrt{h^{2}}$$

where $h^{2}$ is the heritability of the trait. Estimates of $h^{2}$ used to compute r_adj_ were those reported in [13].

Classification of the phenotype for MORT was achieved using several classifiers. These included the EBV_FULL_ for MORT, the genomic predictions of the phenotype for MORT and those of the EBV_FULL_ of all the investigated traits provided by the Bayesian models and the RF algorithm. Moreover, to mimic a scenario of “traditional” selective breeding where only full- and half-sibs of breeding candidates are enrolled in NNV challenge tests, we also considered—as additional classifiers of MORT—the estimated EBV when omitting the phenotype of the individual (EBV_FH_). Hence, in each CV, we omitted the phenotypic information of the animals of the test set from the data, predicted the EBV_FH_ for the investigated traits for all the animals following the methodology described in Section 2.5 and assessed the classification of the phenotype for VNN MORT using the same metrics computed for the other classifiers.

Three metrics were used to evaluate the performance in classifying the phenotype for VNN MORT: the area under the receiving operating characteristics curve (AUC), which describes the natural trade-off in classification between sensitivity (true positive rate) and specificity (true negative rate), the classification accuracy (ACC) and the Matthews correlation coefficient (MCC).

The AUC is the most appropriate metric to evaluate the model performance in classification [45]. This metric measures the ability of a classifier to discriminate the two categories of MORT. An AUC value equal to 0.5 indicates that the performance of the classifier is comparable to that of a classification strategy based on randomly guessing a class, whereas a value of 1 can be obtained when the classifier has a perfect class separation capacity. Hence, an AUC of greater than 0.5 is expected when the classifier performs better than random classification. Further details on receiving operating characteristic (ROC) curves and their use can be found in [46].

The ACC is a measure of the proportion of correctly classified samples and was computed as follows:

(9) $$\mathrm{ACC}=\frac{\mathrm{TP}+\mathrm{TN}}{n}$$

where TP and TN are the number of correctly identified positive (true positives) and negative (true negatives) samples, respectively, and n is the total number of classified samples.

The Matthews correlation coefficient [47] is the correlation coefficient between the observed and predicted binary classifications, which measures the quality of the classification. Such a correlation was computed as follows:

(10) $$\mathrm{MCC}=\frac{\left(\mathrm{TP}\times \mathrm{TN}\right)-\left(\mathrm{FP}\times \mathrm{FN}\right)}{\sqrt{\left(\mathrm{TP}+\mathrm{FP}\right)\left(\mathrm{TP}+\mathrm{FN}\right)\left(\mathrm{TN}+\mathrm{FP}\right)\left(\mathrm{TN}+\mathrm{FN}\right)}}$$

where TP is the number of true positives, TN is the number of true negatives, FP is the number of false positives and FN is the number of false negatives. The coefficient ranges from −1 (total disagreement between the predicted and observed classes) to +1 (perfect prediction). A value of 0 indicates that the classification performance is no better than a random classification. All three metrics were computed in R using the package ROCR [48].

An additional validation procedure, focused on the parents of the challenge-tested fish, was arranged to mimic a real genomic selection scenario where the genomic prediction equations are obtained by training models with information from a reference population that does not include the progeny of the animals to be predicted. We will refer to this procedure as leave-one-family-out (LOFO) validation. In such a procedure, the parents of the experimental fish are used one at a time as an independent test set. When we carried this out, initially, the EBV_FULL_, predicted according to the methodology described in Section 2.5, were retrieved for the parents of the challenge-tested fish. A genomic prediction equation was obtained for each investigated trait by training the BRR model on the EBV_FULL_ of all animals except the offspring of the sire (dam) used as a test set. The EBV_FULL_ of the parent in the test set was then predicted from the parent genotypes by making use of the genomic prediction equation. This was replicated as many times as the number of parents. The predictions and EBV_FULL_ for each parent were saved, and at the end of the LOFO, aggregated with those of the other parents to compute, as a measure of prediction accuracy, the Pearson product-moment correlation (*r*) between the EBV_FULL_ and the genomic-predicted EBV_FULL_.

## 3. Results

### 3.1. Genome-Wide Association Study

**Figure 1:**
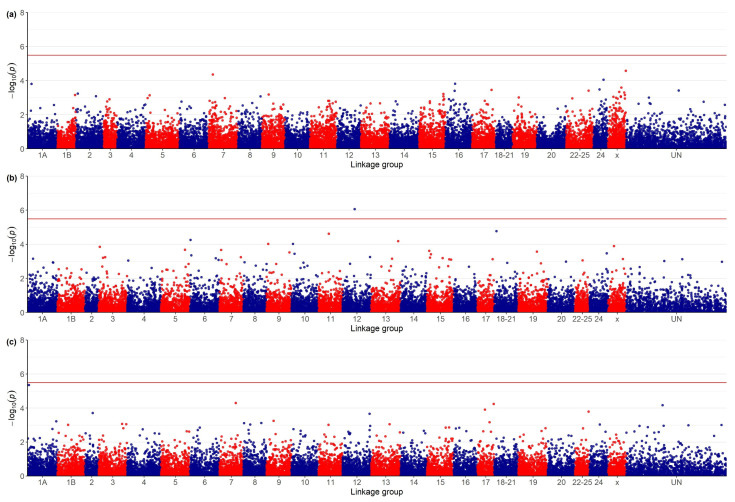
Manhattan plots for genome-wide associations between genotypes at 16,075 SNP and: (**a**) VNN post-challenge mortality, (**b**) square root of serum cortisol concentration (ng^0.5^/^mL0.5^) and (**c**) antibody titer (sample-to-positive ratio of the optical density (OD) values, 450 nm). The red line indicates the genome-wide significance threshold *p* = 0.05/N (N = total number of SNP) after Bonferroni correction and −log_10_ transformation.

The genome-wide significance threshold after Bonferroni correction and after −log_10_ transformation was 5.51. The genome-wide association study failed to identify genome regions exceeding the statistical significance threshold (Figure 1a) and possible associations between MORT and SNP genotypes. For SRHC, the GWAS revealed a single region, belonging to linkage group 12, which exceeded the genome-wide significance threshold and another marker (linkage group 18–21) approaching the significance cut-off (Figure 1b). For AT, one marker in linkage group 1A exhibited a *p*-value very close to the genome-wide significance threshold (Figure 1c).

### 3.2. Genomic Prediction and Classification

#### 3.2.1. Predictive Ability of Genomic Models

In general, the Bayesian models exhibited slightly greater prediction accuracies than the RF algorithm (Table 1). When predicting the phenotype of a trait, the accuracies of the three Bayesian genomic models were very similar. Such accuracies were higher for BW (*r* = 0.38) than for SRHC (*r* = 0.21) and AT (*r* = 0.26). The prediction accuracy of the RF algorithm exhibited a similar pattern, with greater accuracy for BW (*r* = 0.35) than for SRHC and AT (*r* = 0.19 and *r* = 0.24, respectively). The accuracies adjusted for the square root of the trait heritability were moderate and similar across traits (0.50, 0.47 and 0.42 for BW, SRHC and AT, respectively).

As expected, when the models were trained on the EBV_FULL_ of the traits as pseudo-phenotypes, the accuracies increased in comparison with those obtained with models trained on the trait phenotype. For the Bayesian models, the predictive accuracy increased to a similar extent across models. The EBV_FULL_ predicted with the largest accuracy were those of MORT and SRHC (*r* = 0.90). The prediction accuracies were somewhat lower for EBV_FULL_ of BW (*r* = 0.71) and AT (*r* = 0.79). The accuracy of the RF algorithm in predicting the EBV_FULL_ was greater for MORT (*r* = 0.88) and SRHC (*r* = 0.86) than for AT (*r* = 0.76) and BW (*r* = 0.68).

The genomic-predicted EBV_FULL_ were correlated with the observed trait phenotypes (data not presented in tables). Such correlations were greater than those between the observed and genomic-predicted phenotypes for both SRHC and AT, with estimates of *r* ranging from 0.22 to 0.25 and 0.32 to 0.36, respectively.

#### 3.2.2. Leave-One-Family-Out Validation

**Figure 2:**
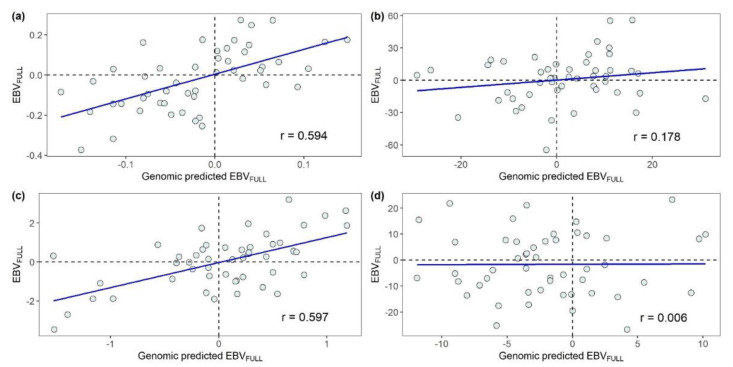
Relationships between the estimated breeding values (EBV_FULL_) and their genomic predictions obtained for the parents of the challenge-tested fish in the leave-one-family-out validation procedure: (**a**) mortality, (**b**) body weight, (**c**) square root of serum cortisol concentration and (**d**) antibody titer. *r*: Pearson product-moment correlation.

Scatterplots of the relationships between the EBV_FULL_ and its genomic predictions obtained in the LOFO validation procedure for the 49 parents of the challenge-tested fish are presented in Figure 2. The prediction accuracy achieved in the LOFO validation procedure was lower in comparison with the accuracy obtained in the set of five-fold random CVs. Large differences in the prediction accuracy were detected across traits. The traits for which the EBV_FULL_ were predicted with the largest accuracy were SRHC (*r* = 0.597; Figure 2c) and MORT (*r* = 0.594; Figure 2a). The prediction accuracy of the EBV_FULL_ for BW was remarkably lower (*r* = 0.178; Figure 2b) and that for AT was so low as to be trivial (*r* = 0.006; Figure 2d).

#### 3.2.3. Performance of Classifiers of Viral Nervous Necrosis Mortality

**Figure 3:**
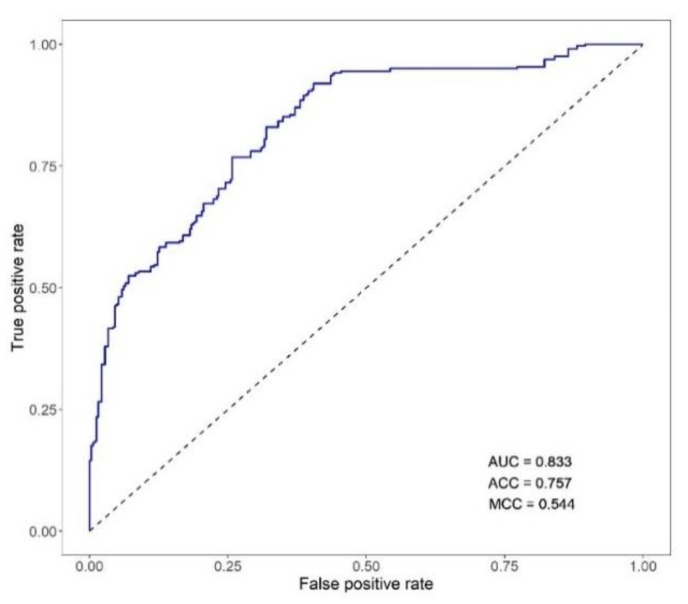
ROC curve for the classification of VNN mortality based on the estimated breeding values of the trait (EBV_FULL_: breeding value estimated using the animal phenotype and the phenotypes of its full- and half-sibs). AUC: area under the ROC curve; ACC: classification accuracy computed as (true positives + true negatives)/number of samples; MCC: Matthews correlation coefficient.

When used as classifiers of the individual phenotype of mortality, the breeding values (EBV_FULL_) for MORT exhibited an AUC of 0.833, ACC of 0.757 and MCC of 0.544 (Figure 3).

**Figure A1:**
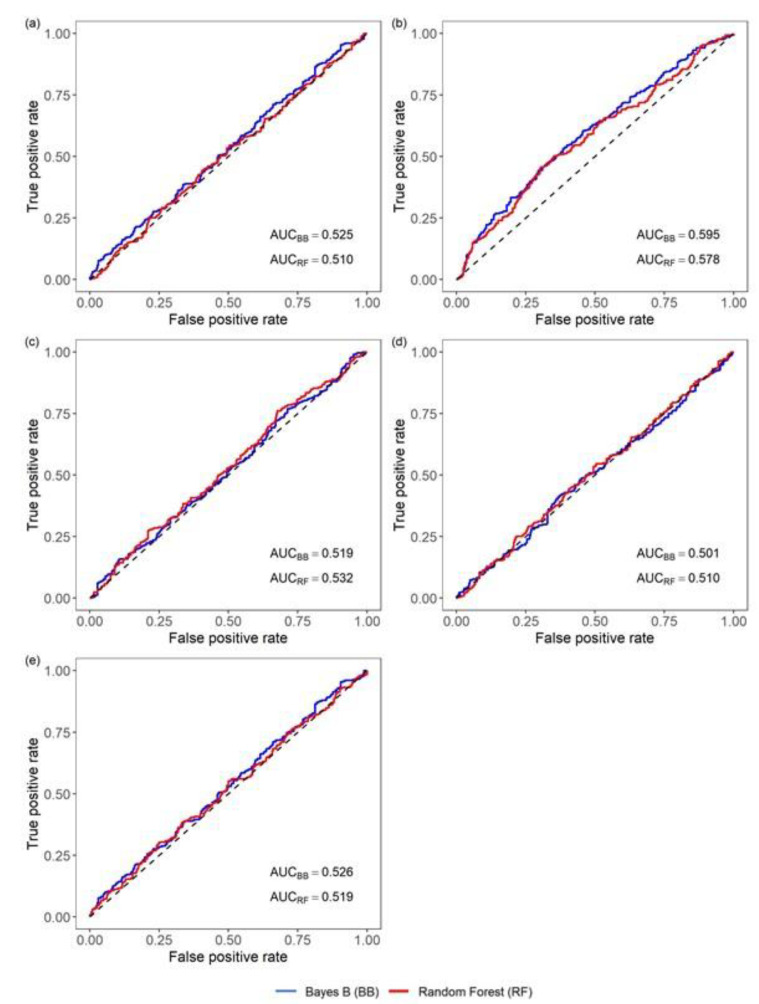
ROC curves for the classification of VNN mortality provided by the genomic predictions of (**a**) phenotype for mortality; (**b**) EBV_FULL_ for mortality; (**c**) EBV_FULL_ for body weight; (**d**) EBV_FULL_ for the square root of serum cortisol concentration; (**e**) EBV_FULL_ for antibody titer (EBV_FULL_: breeding value estimated using the animal phenotype and the phenotypes of its full- and half-sibs). In the figure, the Bayesian model producing the genomic predictions with the best classification performance (Bayes B, BB) is compared with the Random Forest algorithm (RF); the value of the area under the ROC curve (AUC) is reported for both the BB and RF curves.

Classification of the observed MORT using the genomic prediction of the phenotype for MORT as a classifier resulted in unsatisfactory classification performance for all methods (Table 2; Figure A1).

The average AUC was very close to the value expected for this metric (AUC = 0.50) when randomly guessing the classes was used as a classification strategy. The ACC (0.52) and MCC (0.07) metrics also indicated poor classification performance.

When MORT classification was performed using the genomic predictions of the EBV_FULL_ for MORT, the metrics were more favorable (AUC = 0.59; ACC = 0.58; MCC = 0.16), indicating that the genomic classifier contributed an added value to the classification of mortality. The results were consistent across Bayesian models and RF, although the performance of RF was slightly poorer (Table 2).

Using the genomic predictions of the EBV_FULL_ for BW, SRHC and AT as classifiers of VNN mortality led to classifications of poor performance and less favorable metrics than those obtained for the classification based on the genomic-predicted EBV_FULL_ for MORT (Table 2; Figure A1).

**Figure A2:**
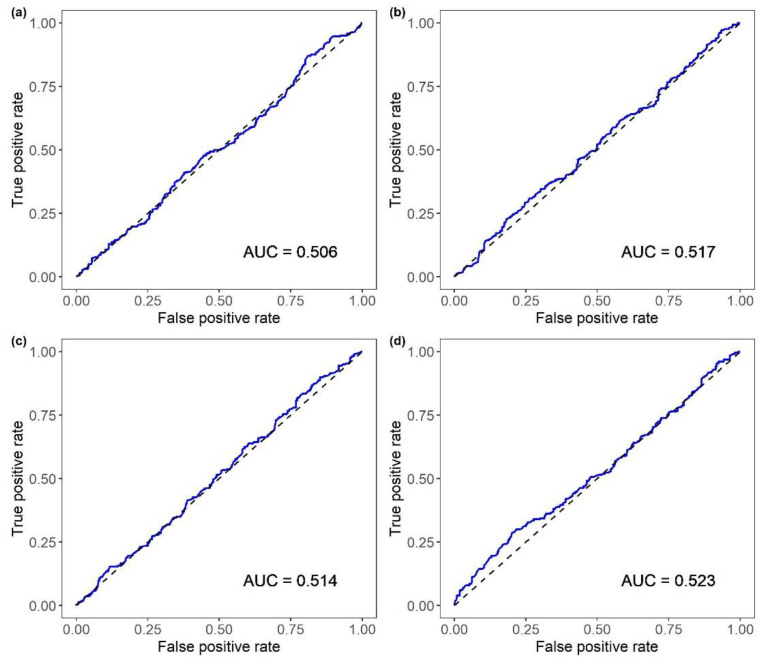
ROC curves for the classification of VNN mortality provided by breeding values estimated using only the information of full- and half-sibs of the individual (EBV_FH_) for (**a**) mortality, (**b**) body weight, (**c**) square root of serum cortisol concentration and (**d**) antibody titer. AUC = area under the ROC curve.

The classification based on the genomic-predicted EBV_FULL_ for BW or AT resulted in an AUC of 0.52, whereas that based on the genomic-predicted EBV_FULL_ for SRHC had an AUC of 0.5. The ACC and MCC were similar across traits, ranging from 0.51 to 0.52 and from 0.04 to 0.07, respectively. Again, the metrics were consistent across Bayesian models and RF. Use of the EBV_FH_—computed using only information on the full- and half-sibs of the individual, as would occur in testing programs based on NNV challenge tests—to classify the VNN MORT led to poor classifications (Table 2; Figure A2). The AUC, ACC and MCC for MORT EBV_FH_ as a classifier were 0.50, 0.53 and 0.09, respectively. Using the EBV_FH_ for BW, SRHC and AT in the classification of MORT led to similar outputs and results comparable to those when randomly guessing the classes.

## 4. Discussion

In this study, we used a genome-wide SNP panel for European sea bass, generated by applying a 2b-RAD sequencing approach, to detect genomic regions associated with the phenotypic variation in MORT, BW, SRHC and AT against NNV and to develop genomic predictions of the phenotype or the EBV for the investigated traits.

For the first time, we performed a GWAS to test the association between SNPs and SRHC or AT in European sea bass. Both the SRHC and AT were associated with only one significant, genome-wide SNP. The reasons for not detecting additional genomic regions exhibiting small effects might be the low statistical power of single-marker GWAS [49], the moderate density of the SNP panel and the genetic complexity of the traits, as evidenced for example in the rainbow trout where several SNPs were significantly associated with the cortisol response to crowding [50].

For VNN mortality, the GWAS failed to identify genome regions exhibiting significant associations with the trait. The lack of significant results may again be explained by the low statistical power of the method [49] and the complex polygenic nature of disease resistance, for which low-to-moderate effects of many genomic regions might be involved in the genetic determinism of the trait [11]. Recent studies on European sea bass reportedly identified significant, genome-wide QTL in different genomic regions, such as linkage groups 3, 20 [2] and 12 [14]. Those studies were based on data from experimental populations three times as large as the experimental population used in our study, and in the case of [14], used a panel of ~40,000 SNP markers.

Genomic selection is a recent field of investigation for aquaculture species, and estimates of the predictive accuracy of genomic models are scarce in the literature. Moreover, to our knowledge, this is the first study to have investigated the performance of genomic models applied to the prediction of traits such as SRHC and AT. The accuracy of the investigated genomic predictions of the phenotype for BW, SRHC and AT ranged from 0.25 to 0.38. The genomic information enabled the additive genetic component of the phenotype to be predicted, which for the investigated traits, accounted for 14 to 57% of the observed phenotypic variation [13]. The upper limit of accuracy attainable when predicting the individual phenotype with a genomic model is the square root of the trait heritability. The estimated accuracies (*r*_adj_) for BW, SRHC and AT were favorable in our study, at 50, 47 and 42%, respectively, of the maximum attainable accuracy. Studies focused on the common carp [4] and Atlantic salmon [51] reported accuracy in the prediction of the additive genetic component of the phenotype ranging from 0.60 to 0.70 for weight and length in juvenile fish or for weight and length measurements taken approximately one year post-hatching, respectively. For the harvest weight in channel catfish, the predictive ability—measured as the correlation between genomic-predicted EBV and phenotypes—was reported as 0.37 [52], while for Nile tilapia, the predictive accuracy of the body weight at about 238 d post-hatching using a GBLUP model was 0.29 [53].

The results obtained for genomic prediction of continuous traits are encouraging in the context of aquaculture production, suggesting that selective breeding approaches using genomic information are of great value in enhancing the aforementioned traits, and become even more important for traits like SRHC and AT, where routine individual phenotyping is a critical issue for various reasons (e.g., practical experimental difficulties, costs and time required).

The EBV_FULL_ for MORT was the best classifier of the observed MORT (Figure 3). In a traditional breeding program where the assessment of the genetic merit of the breeding candidates relies on routine challenge tests, the EBV_FULL_ are not available because the breeding candidates cannot be subjected to NNV infection. Selection is based on estimated breeding values predicted from the phenotypic information of challenged full- and half-sibs families (EBV_FH_), with limitations due to utilizing only the between-family genetic variation [8]. Likewise, for SRHC and AT, difficulties in large-scale phenotyping may restrict the availability of the EBV_FULL_. Though training genomic models on estimated breeding values as pseudo-phenotypes is not as common as for terrestrial species, the development of genomic tools providing accurate predictions of the EBV_FULL_ for MORT and other traits can increase the selection efficiency in fish breeding, thus adding value to selective breeding programs in the aquaculture industry. Since moderate genetic correlations between MORT and BW or AT were estimated in a previous study [13] on the same data, we also investigated the performance of the genomic predictions of the EBV_FULL_ for traits correlated with MORT when used as classifiers of the observed mortality.

The genomic prediction of the EBV_FULL_ led to much greater accuracies than those observed in the prediction of the trait phenotypes; the accuracies were very high for MORT and SRHC and moderately high for BW and AT. Moreover, the correlations between the observed phenotypes and the genomic predictions of the EBV_FULL_ were greater when compared to those between the observed and predicted phenotypes for both SRHC (*r* between the observed phenotypes and genomic predictions of the EBV_FULL_ ranging from 0.22 to 0.25) and AT (*r* ranging from 0.32 to 0.36).

However, the accuracies provided by random k-fold cross-validation are optimistic because in a real genomic selection scenario, the prediction of the genetic merit of future breeding candidates is obtained from data for a reference population that does not include close relatives (e.g., progeny) of the animals to be predicted. To obtain more realistic insights and for strict validation, we developed a LOFO procedure, using the EBV_FULL_ as pseudo-phenotypes. As expected, the LOFO accuracies decreased in comparison with those estimated in the random five-fold cross-validation, but to different extents across traits. For MORT and SRHC, the accuracy remained satisfactory. This is of great interest in the context of application, particularly when considering phenotyping difficulties and complexity for such traits. Omitting the progeny information in the training of the genomic models seems not to affect the accuracy of the genomic-predicted breeding values of the parents, and this means that in a real selection scenario applied to commercial populations, where it is likely that the genomic models are trained using a reference population consisting of distant relatives of the animals to be predicted, the predictive performances of such models would be still acceptable. Conversely, the correlations between the observed and predicted EBV_FULL_ dropped remarkably for BW and AT (−74.9 and −99.2%, respectively). Such trait-specific differences between the LOFO and random validation results are not easy to interpret. It could be argued that factors affecting the accuracy of genomic prediction—like the degree of genomic similarity between the reference (training) and testing (validation) populations, the size and composition of the reference population and the density of the marker array [54]—could have had an impact that depends on trait-specific features. As evidenced by examining the genomic relationships (data not reported), the parents of the challenged fish were weakly related, and accordingly, the relationship between each parent and the animals of the training set used in the LOFO procedure was weak. Hence, in the LOFO procedure, the training of the genomic model used to predict the breeding value of a parent was based on data of animals weakly related to the target individual. When investigating the factors that affect the prediction accuracy of GBLUP (equivalent to the BRR method in our study) in the analysis of human data, de los Campos et al. [55] pointed out that the genomic relationships between the training set used for model building and the testing set to which the model was applied for prediction, and the extent to which marker-derived genomic relationships reflect the patterns of realized genetic relationships at unobserved causal loci underlying complex traits, play a key role in affecting the prediction accuracy of the phenotype. In a study conducted on Atlantic salmon for host resistance to sea lice [5], a consistent decrease in prediction accuracy was observed in several validation sessions where the genetic distance between the training and validation sets was gradually increased. In the five-fold random cross-validations of that study, the prediction accuracy was initially 0.60. Then, it decreased to 0.35–0.50 when different full-sib families were used for training and validation, and it reached 0.05–0.11 when two different populations were used separately, one as the training set and the other as the validation set.

Under perfect linkage disequilibrium between markers and causal loci, the prediction accuracy has an upper bound that depends on the trait heritability and the realized genomic relationships between the training and testing sets [55]. With unrelated or weakly related individuals, which was a feature of the LOFO procedure, the genomic relationships are null or small, but it is still possible to reach a high prediction accuracy, relative to the theoretical upper bound, by enlarging the size of the training population. In our study, the size of the investigated sample (N = 652) was limited by the body size of the challenged fish (146.4 g), which was unusual when compared with that of previous investigations on sea bass or other species [2,15,56] where challenge tests were performed using young juveniles with a body weight not exceeding 20 g.

Relative to the maximum accuracy under perfect linkage disequilibrium, the prediction accuracy under imperfect linkage disequilibrium decreases to an extent that depends on the regression of the realized genomic relationships at the markers on those at the causal genes [55]. Hence, the prediction accuracy can be penalized by the inability of the markers to precisely describe the genetic relationships at causal loci. Such an effect is, however, influenced by specific features of the investigated trait and the density of the marker panel. Weakly related individuals do not share long haplotypes, and high-density markers arrays are needed to capture the genomic similarity between animals [57]. The marker density critical cut-off may be determined by the specific genetic architecture of the trait. As discussed by Kriaridou et al. [58], the selection and exploitation of variants with a direct biological effect on the trait of interest might overcome the drawback of unsatisfactory genomic predictive accuracies due to low-density marker arrays and distantly related individuals. Yet, this requires a vast effort in data collection (genotypes and phenotypes of large reference populations) and functional genomic analyses [59,60].

Using the genomic prediction of the EBV_FULL_ for MORT to classify the observed phenotype of MORT resulted in better classification performance, as measured by the AUC, in comparison with classification based on genomic predictions of the phenotype. The AUC was lower than that (AUC = 0.70) reported by Palaiokostas et al. [2], who used the genomic prediction of the phenotype for VNN resistance to classify the observed survival in a challenge test where sea bass of small sizes were infected by immersion. Such inconsistencies across the two studies may be ascribed to the different nature of resistance assessed, based on the methods of infection adopted in the challenge test. In our study, due to the body size of the experimental fish, virus infection was carried out through injection. The probability of surviving was then influenced by the responses of specific components of the immune system. When using immersion, the outcome of the test is affected by a greater variety of defense mechanisms, which involve unspecific responses by, for instance, mechanical barriers, mucus and other components. This was partly confirmed by the magnitude of the estimated heritability of VNN resistance, which in [2], was approximately twice that detected in our experimental population [13]. Hence, the observed VNN resistance phenotypes in the two studies may actually be observations of different traits.

The unsatisfactory performances exhibited by both the genomic-predicted EBV for continuous traits and EBV_FH_ in the classification of MORT might be explained by the sample size of our experimental population, or by “true” genetic correlations that are weaker than the estimates. The sample size of the reference population used to estimate the allele substitution effects is a major factor in the prediction accuracy, and it also plays a key role in the precision of the estimated genetic correlation between traits. Increasing the number of genotyped animals has been proven to enhance the prediction accuracy [6,61]. The use of EBV_FH_ as a classifier of MORT has a considerable drawback: each member of a full- or half-sib family has an identical EBV_FH_, implying that there is no distinction between members [62] and no possibility of ranking individuals within families [21,63].

## 5. Conclusions

The results of this study indicate that genomic predictions of the estimated breeding values can be used in selective breeding for VNN resistance and response to stressors. This removes the need to phenotype breeding candidates or their relatives, which is necessary in traditional breeding programs. Despite the limited size of the experimental population, which suggests that further investigations are required, the accuracy of the classification of VNN mortality and the prediction of the stress response, as ensured by the genomic prediction models, was greater than that attainable when the genetic merit of breeding candidates is estimated from sibs information, as would occur in selective processes based on challenge tests. Training genomic models with estimated breeding values in place of phenotypes increases the accuracy in classifying or predicting the trait phenotype. The sensitivity of the results of this study to the characteristics of the adopted validation procedure raises questions about the suitability of random cross-validation procedures when the development of genomic models is envisaged in the context of genomic selection. Experimental populations to be subjected to NNV challenge are generated by full or incomplete factorial mating designs that include close relatives. Family linkage disequilibrium may lead to optimistic results when the accuracy of genomic predictions is evaluated on the basis of random cross-validation procedures. The impact of the method of infection used in NNV challenge tests on the type of and variation in the infection response observed should be elucidated in future studies on the genetic background of VNN resistance in European sea bass.

## Figures and Tables

**Table 1:** Average accuracy (SD) of predictions of the phenotype or EBV_FULL_ for the investigated traits provided by the Bayesian models and the Random Forest algorithm in 16 independent five-fold cross-validations.

Trait ^1^	Method ^2^	Prediction of ^3^		
		Phenotype		EBV_FULL_
		*r*	*r*_adj_	*r*
MORT	BB	-	-	0.899 (0.002)
	BC	-	-	0.899 (0.002)
	BRR	-	-	0.899 (0.002)
	RF	-	-	0.875 (0.002)
BW	BB	0.384 (0.016)	0.508 (0.021)	0.710 (0.009)
	BC	0.385 (0.016)	0.509 (0.021)	0.710 (0.009)
	BRR	0.385 (0.016)	0.509 (0.021)	0.710 (0.009)
	RF	0.350 (0.017)	0.464 (0.023)	0.679 (0.010)
SRHC	BB	0.207 (0.017)	0.475 (0.040)	0.900 (0.002)
	BC	0.208 (0.017)	0.479 (0.038)	0.901 (0.003)
	BRR	0.209 (0.017)	0.481 (0.038)	0.901 (0.003)
	RF	0.193 (0.016)	0.444 (0.037)	0.861 (0.002)
AT	BB	0.254 (0.016)	0.426 (0.026)	0.788 (0.005)
	BC	0.256 (0.016)	0.429 (0.027)	0.788 (0.005)
	BRR	0.257 (0.017)	0.430 (0.028)	0.787 (0.005)
	RF	0.243 (0.016)	0.406 (0.027)	0.761 (0.003)

^1^ MORT: post-challenge mortality (0: alive, 1: dead), BW: body weight (g) at 548 d post-hatching, SRHC: square root of serum cortisol concentration (ng^0.5^/^mL0.5^), AT: antibody titer (sample-to-positive ratio of the optical density (OD) values, 450 nm); ^2^ BB: Bayes B, BC: Bayes C, BRR: Bayesian Ridge Regression, RF: Random Forest; ^3^ EBV_FULL_: breeding value estimated using the animal phenotype and the phenotype of its full- and half-sibs; *r*: correlation between the observed value and the prediction; *r*_adj_: correlation between the observed value and the model prediction adjusted for the square root of the trait heritability.

**Table 2:** Average metrics (SD) of classification performance for different classifiers of VNN mortality in 16 independent five-fold cross-validations.

Classifier ^1^	Method ^2^	Metric ^3^		
		AUC	ACC	MCC
genomic-predicted phenotype for MORT	BB	0.525 (0.024)	0.518 (0.010)	0.074 (0.025)
	BC	0.509 (0.026)	0.526 (0.012)	0.076 (0.026)
	BRR	0.505 (0.026)	0.528 (0.013)	0.079 (0.029)
	RF	0.510 (0.017)	0.521 (0.008)	0.067 (0.023)
genomic-predicted EBV_FULL_ for MORT	BB	0.595 (0.004)	0.579 (0.003)	0.165 (0.006)
	BC	0.595 (0.004)	0.580 (0.004)	0.167 (0.006)
	BRR	0.595 (0.004)	0.579 (0.003)	0.167 (0.006)
	RF	0.578 (0.002)	0.572 (0.003)	0.151 (0.005)
genomic-predicted EBV_FULL_ for BW	BB	0.519 (0.005)	0.510 (0.003)	0.060 (0.016)
	BC	0.519 (0.005)	0.510 (0.002)	0.064 (0.018)
	BRR	0.519 (0.005)	0.510 (0.002)	0.063 (0.017)
	RF	0.532 (0.003)	0.507 (0.001)	0.061 (0.014)
genomic-predicted EBV_FULL_ for SRHC	BB	0.501 (0.001)	0.520 (0.004)	0.066 (0.010)
	BC	0.501 (0.001)	0.520 (0.004)	0.067 (0.009)
	BRR	0.501 (0.001)	0.520 (0.005)	0.067 (0.009)
	RF	0.510 (0.002)	0.512 (0.002)	0.071 (0.012)
genomic-predicted EBV_FULL_ for AT	BB	0.526 (0.004)	0.506 (0.003)	0.036 (0.018)
	BC	0.526 (0.004)	0.507 (0.003)	0.040 (0.017)
	BRR	0.526 (0.004)	0.508 (0.003)	0.041 (0.018)
	RF	0.519 (0.002)	0.506 (0.003)	0.066 (0.011)
EBV_FH_ for MORT	BLUP	0.506 (0.009)	0.526 (0.005)	0.090 (0.024)
EBV_FH_ for BW	BLUP	0.517 (0.004)	0.529 (0.005)	0.093 (0.009)
EBV_FH_ for SRHC	BLUP	0.514 (0.009)	0.525 (0.008)	0.085 (0.017)
EBV_FH_ for AT	BLUP	0.523 (0.005)	0.534 (0.006)	0.106 (0.014)

^1^ Genomic predictions of the phenotype or breeding value, estimated including (EBV_FULL_) or omitting (EBV_FH_) the individual phenotypic information, for post-challenge mortality (MORT), body weight at 548 d post-hatching (BW), square root of serum cortisol concentration (SRHC) and NNV antibody titer (AT). ^2^ BB: Bayes B, BC: Bayes C, BRR: Bayesian Ridge Regression, RF: Random Forest, BLUP: best linear unbiased prediction. ^3^ AUC: area under the ROC curve; ACC: classification accuracy computed as (true positives + true negatives)/number of samples; MCC: Matthews correlation coefficient.

## Data Availability

Restrictions apply to the availability of these data. Data are available from the authors with the permission of Valle Cà Zuliani Società Agricola S.r.l. (Conselice, RA, Italy).
